# The Prevalence and Associated Factors of Impaired Fasting Glucose among Children and Adolescents in Urban China: A Large-Scale Cross-Sectional Study

**DOI:** 10.1155/2024/6701192

**Published:** 2024-03-15

**Authors:** Fenglian Huang, Zongyu Lin, Yeling Lu, Yueqin Zhou, Lewei Zhu, Xiaotong Wang, Yanna Zhu

**Affiliations:** Department of Maternal and Child Health, School of Public Health, Sun Yat-sen University, (No.74 Zhongshan Road II), Guangzhou City, China

## Abstract

This study aimed to investigate the level of fasting plasma glucose (FPG), the prevalence of impaired fasting glucose (IFG), and the associated factors among children and adolescents in urban China. Based on a cross-sectional study conducted in three Chinese metropolises during 2013–2014, this analysis included 7,143 participants aged 7–18 years. Information on demographics, family environment, diet, and physical activity was collected by questionnaires. Anthropometric parameters and blood biochemical indicators were measured. Logistic regression models were applied to assess risk factors of glucose level. Results revealed that the average FPG level was 4.81 ± 0.53 mmol/L, and the prevalence of IFG was 3.3%. Trends of these two variables varied significantly with age increasing (all *p* < 0.001), reaching double peaks at 10–12 and 15–17 years. IFG was positively associated with the male sex, age increasing, obesity, higher triglyceride (TG) levels, and living in northern China. When stratified by sex, family history of diabetes, elevated total cholesterol levels, and higher intake of sugar-sweetened beverages were positively associated with IFG only in females, suggesting these parameters were female-specific risk factors of IFG. We concluded that the prevalence of IFG among children and adolescents aged 7–18 years in urban China was higher than that reported in previous regional studies and was associated with obesity and higher levels of TG. Therefore, sex-specific lifestyle interventions should be provided to promote healthy weight and lipids and stem the upward trend of IFG.

## 1. Introduction

Over the past decades, China has undergone rapid socio-economic and nutritional changes [1], leading to tremendous shifts in lifestyle and environment. Accompanying these changes, factors related to diabetes, such as diet, physical activity, blood lipids, and metabolic disorders, have also suffered transformations, which may lead to a rapid increase in the prevalence of diabetes. Increasing prevalence and cases of diabetes among children and adolescents will place a substantial burden on public health, as diabetes will further develop into adulthood and subsequently lead to morbidity and fatality [2, 3]. The global healthcare spending on diabetes shows an increasing trend and is expected to reach $1 trillion by 2030 [4]. China is also one of the countries most affected by diabetes, with the estimated economic loss projected to reach 450.1 billion RMB by 2030 [5].

Impaired fasting glucose (IFG) represents a prediabetic state characterized by abnormal glucose metabolism that does not meet the diagnostic criteria for diabetes [6, 7]. IFG is becoming increasingly common in children [8] and has received much attention worldwide. Subjects with IFG are at high risk for developing diabetes and cardiovascular diseases [9]. Contrary to the irreversibility of diabetes, IFG is a clinical status that typically does not manifest with overt clinical symptoms and is considered to be reversible [8]. Effective intervention in participants with IFG can delay or even prevent the onset of diabetes [10, 11]. Therefore, it is necessary to early identify and prevent IFG in childhood and adolescence, with the aim of reducing the prevalence, complications, and economic burden of diabetes.

Several countries have reported on the prevalence and associated risk factors of IFG among children and adolescents [8, 12–15]. According to data from the National Health and Nutrition Examination Survey, the prevalence of IFG among U.S. adolescents aged 12–19 years was estimated at 7% in 1999–2000 [12]. This figure increased to 11.1% in 2005–2016 [8], indicating a significant rise in the prevalence of IFG among children and adolescents in the United States. Besides, these studies found that age, male sex, ethnicity, urban residency, family income, obesity/weight status, and blood lipids were significantly associated with IFG [12–15], and particularly, male sex, obesity, and dyslipidemia were important risk factors of IFG [14]. However, corresponding statistics are lacking in China. Based on the National Nutrition and Health Survey of China (2002), a national cross-sectional analysis involving 39,928 participants revealed that the prevalence of diabetes among Chinese children and adolescents aged 5–17 years was 0.19% [16]. Unfortunately, the prevalence of IFG in these participants was not reported. Furthermore, China has lacked data from large-scale epidemiological surveys that can reflect the status of glucose metabolism and related influencing factors in children and adolescents. Regional data showed that the prevalence of IFG among Chinese children and adolescents varied from 0.68% to 3.5% [17–20]. Considering the different research time, population, and screening criteria of these regional surveys, the general status of IFG and associated factors in Chinese children and adolescents is not yet clear.

This study aimed to estimate the fasting plasma glucose (FPG) level and the prevalence of IFG among children and adolescents aged 7–18 years in urban China by analyzing the data from a cross-sectional and nationwide representative study conducted in three major cities in China during 2013–2014 [21]. Moreover, this study investigated important factors related to glucose metabolism, including age, sex, weight status, blood lipids, sociodemographic characteristics, and lifestyle elements such as diet, physical activity, and sugar-sweetened beverage intake. Our data can provide a comprehensive overview of the status of IFG among children and adolescents in urban China and help to find out the most influential factors related to IFG, which can potentially promote the establishment of effective interventions for both IFG and diabetes and consequently reduce the economic burden caused by diabetes.

## 2. Materials and Methods

### 2.1. Study Population

The cross-sectional data were obtained from the baseline survey of a school-based interventional project in urban China during 2013–2014. The detailed study design and sampling method of the project have been described previously [21]. Our study sample was a subset from three metropolises: Guangzhou, Shanghai, and Tianjin. In brief, a multistage stratified cluster sampling method was employed to randomly select several regions from the three major cities. Then, 8–12 schools (primary school : secondary school = 1 : 1) were randomly selected from each region. Within the selected schools, two classes were randomly selected from each grade, and questionnaires were collected from all students. Blood samples were collected only from individuals who had signed the informed consent form for blood collection. Ultimately, 7,143 children and adolescents aged 7–18 years agreed to and completed questionnaires and blood biochemical measurements to be included in the study. The epidemiological study was approved by the Ethics Committee of Sun Yat-sen University and received written informed consents from the participants and their parents.

### 2.2. Sample Size Calculation

The purpose of this cross-sectional study was to estimate the prevalence of IFG as well as its associated factors among children and adolescents aged 7–18 years in urban China. For logistic regression, the formula for calculating the minimum sample size required by the research is *N* = 10 × *k*/*p* [22]. *k* is the number of covariates, estimated to be 10 in this study, and *p* is the minimum percentage of IFG in the population, estimated to be 2.0%. The final calculated sample size is approximately 5,000. Considering a nonresponse rate of 20%, the study eventually recruited more than 7,000 people, meeting the sample size requirement.

### 2.3. Questionnaire Survey

Child-reported and parent-reported questionnaires, which have been testified and validated by a pilot survey, were employed in the study [23]. These questionnaires were developed based on the Information, Motivation, and Behavioral Skills model [24]. Information about demographics (sex, age, and provinces/cities), physical activity (vigorous-intensity activity, moderate-intensity activity, walking and sedentary behaviors), and dietary condition (consumption of sugar-sweetened beverages, vegetables, fruit, and meat) were included in child-reported questionnaires. For the children under the third grade, child-reported questionnaires were completed by their parents. Information about the family environment (including paternal and maternal education background and family history of diabetes) were acquired by parent-reported questionnaires. All questionnaires were interpreted by trained project members with a standardized operation manual.

Information about physical activity was collected by the International Physical Activity Questionnaire (Chinese version, IPAQ) [25], which has been used widely in Chinese population studies and has been tested to be acceptable in validity and reliability [26]. For physical activity, each participant was asked to self-report the number of days and hours/minutes per day of daily sedentary behavior, walking, vigorous, and moderate physical activity over the last week. Parental educational background contained three levels: primary or less, secondary, and university or above. A family history of diabetes was defined as at least one family member within three generations suffered from diabetes. To investigate sugared beverages consumption and dietary condition, the following questions were raised: “How many cups of sugared beverages did you drink each day last week (sugar-sweetened beverages contained carbonated beverages and sugar-sweetened milk drinks, a cup is about 250 mL)?”.

Taking the Qinling–Huaihe as the boundary, the three provinces/cities were further artificially divided into the northern regions (Tianjin and Shanghai) and the southern regions (Guangzhou).

### 2.4. BMI and Weight Status

Height and weight were measured by well-trained and experienced doctors and nurses with standardized procedures. The height was measured using a fixed stadiometer (Yilian TZG, Jiangsu, PRC) to the nearest 0.1 cm. The weight was measured using a lever scale (Hengxing RGT-140, Jiangsu, PRC) to the nearest 0.1 kg. All measurements were taken twice for each participant, and the average values were calculated. BMI was calculated as weight in kilograms divided by the square of height in meters. Percentiles for BMI were determined to be specific to sex and month of age. Students were classified into wasting, normal weight, overweight, and obesity groups. According to the BMI reference norm proposed by the Group of China Obesity Task Force in 2004, participants with an age- and sex-specific 85th ≤ BMI < 95th percentile were defined as overweight, while those with a BMI ≥ 95th percentile were defined as obesity [27]. Wasting was also defined using age- and sex-specific BMI in reference to the Screening Standard for Malnutrition of School-age Children and Adolescents (WS/T 456-2014) published by the National Health and Family Planning Commission of the People's Republic of China [28].

### 2.5. Biochemical Analyses

After an overnight fast for at least 12 hr, venous blood samples (5 mL) were taken from each participant's antecubital vein and were collected into EDTA vacuum tubes. Thereafter, blood samples were centrifuged at 3,000 r/min and then stored at −80°C until being analyzed. All biochemical indicators were tested at a biomedical analysis company accredited by Peking University. FPG, total cholesterol (TC), triglyceride (TG), high-density cholesterol (HDL-C), and low-density cholesterol (LDL-C) were formally measured.

### 2.6. Study-Outcome Definitions

The average FPG and the prevalence of IFG were the outcomes of this study. According to the American Diabetes Association (ADA) criteria [29], glucose <5.6 mmol/L was defined as normal FPG, while the concentration of FPG between 100 and 125 mg/dL (5.6–6.9 mmol/L) was classified as IFG.

### 2.7. Statistical Analyses

Data were analyzed using the Social Science Statistics Package (SPSS) (version 21.0, SPSS, Chicago, IL, USA). Continuous variables, such as height, weight, BMI, blood sample outcomes, dietary intake, and duration of physical activity, were analyzed using an independent *t*-test or analysis of variance. Categorical variables, such as sex, geographical location, weight status, family history of diabetes, and parental education level, were analyzed using the Chi-square test. It should be noted that we categorized the distribution of weight status into four levels when describing the basic characteristics of study participants, but we did not include the level of wasting in subsequent analysis because this study mainly focused on the effects of overweight and obesity on glucose metabolism. Trend analysis was carried out by one-way ANOVA to examine the trend of fasting glucose with increasing age and weight status, i.e., from normal weight to overweight to obesity. Similarly, the Chi-square test was used to examine the trend of IFG prevalence with increasing age and weight status. The odds ratios (ORs) of potential influencing factors (e.g., sex, age, geographical location, family history of diabetes, parental education level, sugar-sweetened beverage intake, weight status, TG, TC, LDL-C, and HDL-C) related to IFG were calculated using univariate and age-adjusted and sex-adjusted multivariable logistic regression. The OR and the 95% confidence interval (CI) were presented as measures of correlation and precision. All *p* values were bilateral, and *p* < 0.05 was used to identify statistical significance.

## 3. Results

### 3.1. Basic Characteristics of the Study Population

The basic characteristics of the participants are shown in Table 1, including demographics, anthropometric parameters, biochemical indicators, duration of physical activity, and dietary intake. Significant differences were found in anthropometric parameters, including age, height, weight, BMI, BMI *z*-score, and weight status, between males and females (all *p* < 0.001). Additionally, there were significant differences in the levels of blood lipids, including TC, TG, HDL-C, LDL-C, and FPG, between males and females (all *p* < 0.001).

### 3.2. Status of FPG and IFG According to Sociodemographics

As presented in Table 2, the mean FPG was 4.81 ± 0.53 mmol/L, which was higher in males than in females (4.87 ± 0.55 vs. 4.74 ± 0.49 mmol/L, *p* < 0.001). The prevalence of IFG in total participants was 3.3%, and it was higher in males than in females (4.6% vs. 2.0%, *p* < 0.001). In addition, there were significant differences in mean FPG and prevalence of IFG depending on geographical conditions. These two parameters were significantly higher in participants living in the northern area than those living in the southern area (all *p* < 0.001). Significant differences in those two parameters were also observed in different provinces/cities (all *p* < 0.001).

### 3.3. Status of FPG and IFG According to Age and Sex

**Figure 1:**
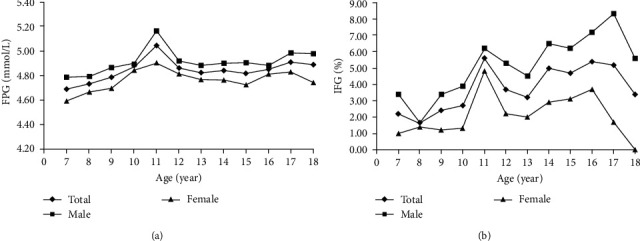
Status of fasting glucose according to age and sex. Panel (a) shows the average levels of FPG (mmol/L), and Panel (b) shows the prevalence (%) of IFG according to age and sex. Trend analysis was carried out by one-way ANOVA or Chi-square test to examine the trend of average FPG or IFG prevalence with increasing age, all *p* for trend <0.001. *Note*. FPG, fasting plasma glucose; IFG, impaired fasting glucose.

As shown in Figure 1(a), the mean value of FPG showed a significant trend with increasing age (*p* for trend <0.001). At the age of 7–18, FPG showed double peaks, which occurred at 10–12 years old (5.04 ± 0.90 mmol/L) and 15–17 years old (4.91 ± 0.51 mmol/L). The highest levels of FPG for males and females were 5.16 ± 1.14 and 4.90 ± 0.43 mmol/L, respectively, both occurring at the age of 11; and the lowest levels for males and females were 4.79 ± 0.44 and 4.59 ± 0.41 mmol/L, respectively, both at the age of 7. In general, the average FPG of males was significantly higher than that of females at all ages.

As shown in Figure 1(b), the prevalence of IFG also showed a significant trend with increasing age (*p* for trend <0.001). The overall prevalence of IFG reached peaks of 5.6% at 10–12 years old and 5.4% at 15–17 years old. For males, the highest prevalence of IFG was 8.3%, occurring at the age of 17, and the lowest was 1.7% at the age of 8. For females, the highest was 4.8% at the age of 11, and there was no prevalence (0%) at the age of 18, which is possibly due to the limited size of the 18-year-old group in this study (*n* = 29; 11 females). Compared with females, the prevalence of IFG in males was higher in all age groups (*p* < 0.001).

### 3.4. Association of Fasting Glucose with Weight Status and Blood Lipids

As can be seen from Table 3, the prevalence of IFG in the total population, females, and participants with secondary parental educational levels showed a significant trend with increasing weight status, i.e., from normal weight to overweight to obesity (all *p* for trend <0.05).  Table 4 shows that participants with IFG had higher levels of TG than normal participants (all *p* < 0.05), and this finding still existed in the subgroups of females and participants living in northern China (*p* < 0.05).

### 3.5. Association of Fasting Glucose with Physical Activity and Dietary Intakes

As shown in Table S1 in Supplementary Materials, the intake of sugar-sweetened beverages in participants with IFG was significantly higher than that in normal participants (all *p* < 0.05). No significant difference in physical activity was found between normal participants and IFG subjects.

### 3.6. Influencing Factors of IFG

The results of binary logistic regression (Table 5) showed that male sex (OR = 2.353, 95% CI: 1.774, 3.120), increasing age (OR = 1.122, 95% CI: 1.078, 1.167), living in the northern area (OR = 3.041, 95% CI: 2.113, 4.376), obesity (OR = 1.647, 95% CI: 1.133, 2.395) and higher level of TG (OR = 1.312, 95% CI: 1.087, 1.583) was associated with increased risk of IFG for children and adolescents in urban China (all *p* < 0.05), which still can be observed in the female subgroup. Additionally, family history of diabetes (OR = 1.959, 95% CI: 1.188, 3.231), sugar-sweetened beverages (OR = 1.284, 95% CI: 1.010, 1.632), and higher TC levels (OR = 1.356, 95% CI: 1.022, 1.799) were also related to increased risk of IFG in females. Only increasing age (OR = 1.120, 95% CI: 1.068, 1.174) and living in the northern area (OR = 3.018, 95% CI: 1.959, 4.649) were associated with the increased risk of IFG in males.

## 4. Discussion

Currently, data from developed countries have proven a striking increase in the prevalence of IFG among children and adolescents, but data from developing and underdeveloped countries are still scarce. As one of the largest developing countries in the world, China urgently needs to overview the status of IFG and its associated factors among children and adolescents.

### 4.1. Prevalence of IFG

In the present study, the prevalence of IFG among children and adolescents in urban China was significantly higher than that reported in previous regional studies. A survey of 19,593 participants aged 6–18 years reported that the prevalence of IFG in Beijing (the capital of China) was 1.35% in 2004 [20]. In 2006, a survey of 3,937 adolescents aged 13–18 years in Hebei province of China showed that the prevalence of IFG was 3.5% [19], while this prevalence should actually be much lower in participants aged 7–18 years owing to the fact that IFG is highly prevalent in puberty. Data from 2010 to 2011 in Xinjiang, China, showed that the prevalence of IFG among people under 13 years old was about 0.7% [17]. The present study found that the prevalence of IFG among children and adolescents in urban China was 3.3% in 2013–2014, which provided new population-based evidences for the prevalence of IFG among Chinese children and adolescents and indicated the potential upward trend of IFG prevalence.

The prevalence of IFG among Chinese children and adolescents in our study was lower than that reported in developed countries, including Mexico (18.3%), the United States (13.1%), and the United Kingdom (20%–30%) [8, 13, 15]. The obvious difference in IFG prevalence between urban China and other developed countries can be partly explained by the lower obesity rate among Chinese children and adolescents [30]. However, it cannot be excluded that the different prevalence of IFG in different countries may be related to the different age groups of the research population.

Moreover, the prevalence of IFG in Chinese children and adolescents was found to be far lower than that in Chinese adults. In 2015, a study of 1,495 Chinese adults over the age of 20 reported a 15.5% prevalence of IFG [31], while another survey reported a prevalence of 4.0% among 10,800 middle-aged adults in China [32]. IFG is a precursor status of type 2 diabetes, and previous studies have associated IFG during childhood and adolescence with an increased risk of type 2 diabetes in adulthood [33]. Therefore, despite the lower prevalence of IFG observed in children compared to adults, IFG in childhood should not be overlooked, considering its close relationship with future diabetes.

Males were observed to be at a higher risk of IFG than females, and most studies conducted in China and abroad (e.g., in Korea, Australia, and Saudi Arabia) have revealed this sex preference [14, 34–36]. In our study, the higher prevalence of IFG in males could be attributed to the higher prevalence of obesity among males. However, some studies, such as those conducted in the United States and Jamaica, contradicted our observation [37, 38], which claimed that the prevalence of IFG was higher among females. In addition, a systematic review of 14,721 children in 2013 found no significant sex difference among Swedish diabetic children [39]. This suggests that sex disparities in IFG prevalence found across studies may be owing to racial differences [40].

The prevalence of IFG in the present study varied significantly with age increasing and was higher at the ages of 10–12 and 15–17 years, coinciding with the duration of puberty. The SAUDI-DM Project in Saudi Arabia and an earlier survey in Taiwan also showed that the age of 13–18 years was an important risk factor for IFG [14, 41]. Increasing evidence have suggested that adolescence is closely associated with increased insulin resistance, as proved by other epidemiological studies investigating hyper-insulin-anemia in pubertal-age adolescents [42, 43]. Previous studies have highlighted the importance of early lifestyle interventions in preventing T2DM, impaired glucose tolerance (IGT), and IFG in young healthy individuals [44]. Given the close relationships between puberty and disturbances in glucose and insulin secretion, it is advisable to carry out interventions before adolescence in order to reduce the early onset of IFG and diabetes. The prevalence of IFG declined at the age of 18 years, possibly due to the limited sample size of the 18-year-old group in this study (*n* = 29).

### 4.2. Influencing Factors of IFG

In the present study, we found a significant increase in the prevalence of IFG with increasing weight status, i.e., from normal weight to overweight to obesity, which was consistent with the Swedish cohort study conducted in 2015 [45]. The study in Sweden showed that the prevalence of IFG in obese children was 35.8%, which was higher than that in normal-weight children. A survey of 1,034 Emirati overweight/obese children aged 12–17 years found that the prevalences of prediabetes and diabetes were higher in overweight/obese participants [46]. Similar findings have also been found in adults. In 2015, a cohort study of 24,930 Austrian adults aged 20–40 showed that participants with persisting obesity had the highest IFG risk [47]. Our results may be explained by the previous findings in adults and adolescents; that is, obese participants had a tendency to reduce hepatic insulin sensitivity or had higher levels of peripheral insulin resistance and progressive beta-cell failure [48, 49] that resulted in their high prevalence of diabetes. Over the past 20 years, the obesity rate of children in urban China has risen rapidly [50]. It was estimated that about 30% of males and 16% of females in urban China were overweight or obese [51]. This suggests that a large proportion of Chinese children are at high risk of IFG and diabetes, and this proportion is likely to continue increasing. This also urges the Chinese healthcare system to implement targeted intervention strategies for preventing and delaying the onset and progression of IFG or T2DM.

Our study found that high levels of blood lipids, especially TG, were significantly associated with an increased risk of IFG. This finding was consistent with a previous study that suggested TG to be an important independent risk factor for IFG [14]. Since the abnormal lipid parameters were part of the metabolic syndrome [52], which has been recognized more recently among children and adolescents, this could explain the association between abnormal blood lipids and abnormal glucose metabolism.

It is generally believed that fasting glucose is closely related to lifestyle, especially dietary intake and physical activity [3, 53–54]. In the present study, we found that sugar-sweetened beverages were associated with an increased risk of IFG, which can be explained by its close association with obesity and insulin resistance [3]. However, no significant results were observed in physical activity, a finding that contradicts previous studies [53–54], possibly due to differences in the measuring methods for physical activity and the age discrepancies across the study samples. Unlike previous studies that used accelerometers to assess physical activity in adolescents aged 10–17 [54], our study used a self-report questionnaire to measure physical activity. Thus, recall bias and age differences in the present study could contribute to the discrepancies [55].

Interestingly, our study found that although the prevalence of IFG in females is lower than that in males, the number of risk factors in females was significantly higher than that in males. Although females are more likely to be exposed to the risk factors of IFG due to their family history of diabetes, higher level of TC, and lifestyle preferences, they have a unique glucose regulation mechanism, which is lacking in males to maintain normal FPG levels. Studies found that estrogens have strong antidiabetic actions by increasing insulin secretion and decreasing glucagon secretion in females, enhancing insulin sensitivity and, more generally, exerting protective function on beta cells [56–58]. Moreover, the distinctive distribution of adipose tissue in females compared to males also contributes to the maintenance of glucose homeostasis [59]. Therefore, even though females were susceptible to more risk factors, the prevalence of IFG in females was still lower than that in males, probably due to the effect of estrogen in regulating blood lipids and glucose.

### 4.3. Strengths and Limitations

Compared with prior studies, this study provided an estimate of IFG prevalence among urban population aged 7–18 in China during 2013–2014. For the strengths, a large sample size and wide age span were used in the present study, which provided a representative overview of fasting glucose and its related factors in urban Chinese children and adolescents. In addition, this study considered a variety of factors affecting fasting glucose, including age, sex, geographical location, family history of diabetes, parental education background, weight status, blood lipids, dietary intake, and physical activity. The limitation of the study is that the prevalence of diabetes was not estimated due to the lack of clinical symptom information and repeated measurements of FPG. As the ADA standard has emphasized, in the absence of clinical symptoms of hyperglycemia, the diagnosis of diabetes must be confirmed by repeated testing on different days [29]. Besides, as our study population was limited to three Chinese metropolises, attention should be payed when extrapolating these findings. Finally, employing univariate, age-adjusted, and sex-adjusted multivariable logistic regression, we revealed key factors that were significantly associated with IFG. We suggest that future studies should use multivariate analysis methods that include more comprehensive covariates to deepen the understanding of the interactions between these factors and their complex effects.

## 5. Conclusions

The present study utilizes data from 2013 to 2014 to overview the status of IFG and associated factors among children and adolescents in urban China. Among participants aged 7–18 years, the average level of FPG was 4.81 ± 0.53 mmol/L, and the prevalence of IFG was 3.3%, lower than that in most developed countries but significantly higher than that reported in previous regional studies. IFG was positively associated with the male sex, age increase, obesity, higher TG levels, and living in northern China. When stratified by sex, family history of diabetes, elevated TC levels, and higher intake of sugar-sweetened beverages were positively associated with IFG only in females, suggesting these parameters were female-specific risk factors of IFG. Considering the upward trend of IFG and the huge population base in China, effective measures should be taken in time for early intervention, even if the prevalence of IFG in China is at a low level. Sex-specific lifestyle interventions should be provided to promote healthy weight and lipids, especially before adolescence.

## Figures and Tables

**Table 1:** Basic characteristics of the study population (*N* = 7,143).

Variables	Total	Male	Female	*p* Value ^*∗*^
Demographic				
Sex, *n* (%)^†^				
Male	3,629 (50.8)	—	—	—
Female	3,514 (49.2)	—	—	—
Age (year), M ± SD^‡^	11.33 ± 3.29	10.96 ± 3.12	11.11 ± 3.17	**<0.001**
Age group, *n* (%)^†^				
7−9 years	2,757 (38.6)	1,405 (38.7)	1,352 (38.5)	0.964
10−14 years	2,449 (34.3)	1,239 (34.2)	1,210 (34.3)	
15−18 years	1,937 (27.1)	985 (27.1)	952 (27.2)	
Geographical location, *n* (%)^†^				
Northern area	4,621 (64.7)	2,356 (64.9)	2,265 (64.5)	0.692
Southern area	2,522 (35.3)	1,273 (35.1)	1,249 (35.5)	
Family environment				
Paternal education level, *n* (%)^†^				
None/primary	302 (4.2)	151 (4.2)	151 (4.3)	0.988
Secondary	5,070 (71.0)	2,549 (70.2)	2,521 (71.7)	
University or above	1,297 (18.2)	649 (17.9)	648 (18.4)	
Unknown	474 (6.6)	280 (7.7)	194 (5.5)	
Maternal education level, *n* (%)^†^				
None/primary	502 (7.0)	261 (7.2)	241 (6.9)	0.690
Secondary	5,042 (70.6)	2,522 (69.5)	2,520 (71.7)	
University or above	1,120 (15.7)	559 (15.3)	561 (16.0)	
Unknown	479 (6.7)	287 (2.9)	192 (5.5)	
Family history of diabetes, *n* (%)^†^				
Yes	1,684 (23.6)	839 (23.1)	845 (24.0)	0.931
No	4,339 (60.7)	2,156 (59.4)	2,183 (62.1)	
Unknown	1,121 (15.7)	634 (17.5)	486 (13.8)	
Anthropometric parameters, M ± SD				
Height (cm)^**‡**^	149.3 ± 16.9	151.29 ± 18.65	147.26 ± 14.7	**<0.001**
Weight (kg)^**‡**^	43.9 ± 16.9	46.3 ± 18.8	41.5 ± 14.4	**<0.001**
BMI (kg/m^2^)^**‡**^	19.0 ± 4.17	19.4 ± 4.40	18.5 ± 3.86	**<0.001**
BMI *z*-score	0.00 ± 1.00	0.11 ± 1.01	−0.11 ± 0.93	**<0.001**
Weight status, *n* (%)^†^				
Normal weight	4,755 (66.6)	2,138 (58.9)	2,617 (74.5)	**<0.001**
Overweight	1,115 (15.6)	642 (17.7)	473 (13.5)	
Obesity	771 (10.8)	561 (15.5)	210 (6.0)	
Wasting	268 (3.8)	146 (4.0)	123 (3.5)	
Unknown	234 (3.3)	142 (4.0)	91 (2.6)	
Biochemical indicators, M ± SD				
TC (mmol/L)^**‡**^	4.08 ± 0.79	4.01 ± 0.79	4.15 ± 0.78	**<0.001**
TG (mmol/L)^**‡**^	0.91 ± 0.47	0.89 ± 0.46	0.93 ± 0.48	**<0.001**
HDL (mmol/L)^**‡**^	1.36 ± 0.33	1.35 ± 0.33	1.38 ± 0.33	**<0.001**
LDL (mmol/L)^**‡**^	2.21 ± 0.66	2.17 ± 0.65	2.25 ± 0.66	**<0.001**
FPG (mmol/L)^**‡**^	4.81 ± 0.53	4.87 ± 0.55	4.74 ± 0.49	**<0.001**
Physical activity, M ± SD				
Vigorous intensity activity (min/day)^**‡**^	81.24 ± 88.35	81.59 ± 87.60	80.85 ± 89.20	0.808
Moderate intensity activity (min/day)^**‡**^	76.48 ± 80.58	76.18 ± 78.68	76.78 ± 82.49	0.813
Walking (min/day)^**‡**^	74.50 ± 76.66	75.72 ± 79.61	73.26 ± 73.52	0.231
Sedentary behavior (min/day)^**‡**^	366.55 ± 226.1	361.63 ± 224.53	371.47 ± 229.9	0.090
Dietary conditions (servings/day), M ± SD				
Fruit^**‡**^	1.52 ± 0.87	1.50 ± 0.84	1.53 ± 0.90	0.187
Vegetables^**‡**^	2.01 ± 1.19	2.01 ± 1.20	2.02 ± 1.18	0.659
Meat^**‡**^	1.33 ± 0.88	1.34 ± 0.89	1.32 ± 0.87	0.485
Sugar-sweetened beverages^**‡**^	1.24 ± 0.69	1.26 ± 0.74	1.22 ± 0.64	0.110

*Note*: TC, total cholesterol; TG, triglyceride; HDL-C, high-density cholesterol; LDL-C, low-density cholesterol; FPG, fasting plasma glucose; BMI, body mass index. ^†^Categorical variables. ^‡^Continuous variables.  ^*∗*^Male vs. female, Chi-square test for categorical variables and *t*-test for continuous variables. *P* values <0.05 are presented in bold.

**Table 2:** Different status of FPG^†^ according to sociodemographics (*N* = 7,143).

Characteristics	Mean FPG^‡^ (M ± SD, mmol/L)	*p* Value	IFG^§^*n* (%)	*p* Value
Total participants	4.81 ± 0.53	—	238 (3.3)	—
Sex	—	**<0.001**	—	**<0.001**
Male	4.87 ± 0.55	—	167 (4.6)	—
Female	4.74 ± 0.49	—	71 (2.0)	—
Geographical location				
Qinling–Huaihe as the boundary	—	**<0.001**	—	**<0.001**
Northern area	4.93 ± 0.42	—	203 (4.4)	—
Southern area	4.59 ± 0.62	—	35 (1.4)	—
Provinces/cities	—	**<0.001**	—	**<0.001**
Shanghai	4.92 ± 0.45	—	120 (5.7)	—
Guangzhou	4.59 ± 0.62	—	35 (1.4)	—
Tianjin	4.94 ± 0.39	—	83 (3.3)	—
Paternal education level		**<0.001**		0.071
None/primary	4.95 ± 0.84	—	12 (4.0)	—
Secondary	4.83 ± 0.50	**—**	185 (3.7)	—
University or above	4.68 ± 0.51	**—**	31 (2.4)	—
Maternal education level	—	**<0.001**	—	0.164
None/primary	4.94 ± 0.54	—	19 (3.8)	—
Secondary	4.83 ± 0.52	—	182 (3.6)	—
University or above	4.70 ± 0.54	**—**	28 (2.5)	—

*Note*: FPG, fasting plasma glucose; IFG, impaired fasting glucose. ^†^Status of FPG, including mean FPG and IFG. ^‡^Mean FPG was compared by using *t*-test or one-way ANOVA. ^§^Prevalence of IFG was compared by using Chi-square test. *P* values <0.05 are presented in bold.

**Table 3:** Different status of FPG^†^ stratified by weight status (*N* = 6,875)^‡^.

Groups	Mean FPG (M ± SD, mmol/L)			*p* for trend ^*∗*^	IFG (*n*, %)			*p* for trend ^*∗∗*^
	Normal weight	Overweight	Obesity		Normal weight	Overweight	Obesity	
Total population	4.77 ± 0.52	4.87 ± 0.45	4.91 ± 0.47	0.593	138 (2.9)	39 (3.5)	39 (5.1)	0.054
Male	4.85 ± 0.56	4.92 ± 0.46	4.91 ± 0.47	0.424	95 (4.4)	29 (4.5)	29 (5.2)	0.992
Female	4.72 ± 0.49	4.80 ± 0.42	4.91 ± 0.47	0.706	43 (1.6)	10 (2.1)	10 (4.8)	**0.046**
Northern area	4.90 ± 0.42	4.95 ± 0.38	4.98 ± 0.42	**0.032**	115 (3.8)	36 (4.7)	32 (5.6)	0.082
Southern area	4.56 ± 0.60	4.68 ± 0.52	4.67 ± 0.53	0.967	23 (1.3)	3 (0.9)	7 (3.6)	0.487
Paternal education level								
None/primary	4.95 ± 0.99	4.87 ± 0.39	5.10 ± 033	0.900	9 (4.5)	1 (1.8)	2 (6.7)	0.789
Secondary	4.80 ± 0.48	4.90 ± 0.45	4.94 ± 0.48	0.909	100 (3.0)	34 (4.3)	32 (5.6)	**0.014**
University or above	4.65 ± 0.50	4.76 ± 0.45	4.83 ± 0.48	0.160	21 (3.4)	3 (1.5)	4 (3.3)	0.712
Maternal education level								
None/primary	4.92 ± 0.60	4.96 ± 0.43	5.03 ± 0.37	0.457	12 (3.4)	4 (5.6)	3 (5.9)	0.675
Secondary	4.80 ± 0.51	4.88 ± 0.45	4.94 ± 0.44	0.771	101 (3.0)	29 (3.7)	32 (5.5)	**0.027**
University or above	4.67 ± 0.50	4.80 ± 0.47	4.79 ± 0.66	0.224	18 (2.4)	5 (2.6)	3 (3.3)	0.881

*Note*: FPG, fasting plasma glucose; IFG, impaired fasting glucose. ^†^Status of FPG, including mean FPG and IFG. ^‡^Wasting patients were not included for analysis.  ^*∗∗*^Trend analysis was carried out by Chi-square test to examine the trend of IFG prevalence with increasing weight status. *P* values <0.05 are presented in bold.

**Table 4:** Mean blood lipids in participants with different status of FPG^†^ (*N* = 7,143).

Blood lipids (M ± SD, mmol/L)	Participants with normal FPG	Participants with IFG	*p* Value ^*∗*^
Total population			
TG	0.91 ± 0.47	1.00 ± 0.58	**0.001**
TC	4.08 ± 0.79	4.08 ± 0.73	0.941
LDL-C	2.21 ± 0.66	2.21 ± 0.64	0.937
HDL-C	1.36 ± 0.33	1.34 ± 0.36	0.274
Male			
TG	0.89 ± 0.46	0.93 ± 0.49	0.236
TC	4.01 ± 0.80	3.98 ± 0.69	0.526
LDL-C	2.17 ± 0.65	2.14 ± 0.60	0.516
HDL-C	1.35 ± 0.33	1.35 ± 0.33	0.673
Female			
TG	0.93 ± 0.47	1.16 ± 0.74	**<0.001**
TC	4.15 ± 0.78	4.32 ± 0.77	0.070
LDL-C	2.24 ± 0.66	2.36 ± 0.70	0.145
HDL-C	1.38 ± 0.33	1.35 ± 0.38	0.423
Northern area			
TG	0.92 ± 0.47	1.01 ± 0.60	**0.005**
TC	3.99 ± 0.74	4.07 ± 0.72	0.128
LDL-C	2.13 ± 0.62	2.21 ± 0.63	0.094
HDL-C	1.35 ± 0.32	1.34 ± 0.35	0.450
Southern area			
TG	0.89 ± 0.45	0.92 ± 0.45	0.760
TC	4.25 ± 0.85	4.14 ± 0.81	0.437
LDL-C	2.35 ± 0.69	2.20 ± 0.70	0.207
HDL-C	1.38 ± 0.34	1.35 ± 0.43	0.647

*Note*: FPG, fasting plasma glucose; IFG, impaired fasting glucose; TC, total cholesterol; TG, triglyceride; HDL-C, high-density cholesterol; LDL-C, low-density cholesterol. ^†^Status of FPG, including normal FPG and IFG.  ^*∗*^Participants with IFG vs. participants with normal FPG, analyzed by *t*-test. *P* values <0.05 are presented in bold.

**Table 5:** Association of IFG with related factors (*N* = 7,143).

Variables	Total population		Male		Female	
	OR (95% CI)	*p* Value ^*∗*^	OR (95% CI)	*p* Value ^*∗*^	OR (95% CI)	*p* Value ^*∗*^
Male sex						
Model 1	2.341 (1.767, 3.103)	**<0.001**	—	—	—	—
Model 2	2.353 (1.774, 3.120)	**<0.001**	—	—	—	—
Age, per increase of 1 year						
Model 1	1.121 (1.078, 1.166)	**<0.001**	1.120 (1.068, 1.174)	**<0.001**	1.127 (1.048,1.212)	**<0.001**
Model 2	1.122 (1.078, 1.167)	**<0.001**	—	—	—	—
Living in northern China						
Model 1	3.261 (2.271, 4.684)	**<0.001**	3.194 (2.076,4.914)	**<0.001**	3.430 (1.751,6.717)	**<0.001**
Model 2	3.041 (2.113, 4.376)	**<0.001**	3.018 (1,959,4.649)	**<0.001**	1.105 (1.028,1.188)	**0.007**
Family history of diabetes						
Model 1	1.140 (0.849, 1.532)	0.384	0.842 (0.577, 1.229)	0.373	2.058 (1.250, 3.388)	**0.005**
Model 2	1.116 (0.829, 1.503)	0.468	0.836 (0.572, 1.222)	0.355	1.959 (1.188, 3.231)	**0.008**
Maternal education level is university or above						
Model 1	0.651 (0.360, 1.177)	0.156	0.690 (0.345, 1.379)	0.294	0.594 (0.187, 1.890)	0.378
Model 2	0.673 (0.371, 1.221)	0.193	0.693 (0.346, 1.388)	0.301	0.625 (0.196, 1.992)	0.427
Sugar-sweetened beverage intake per increase of 250 mL						
Model 1	1.181 (1.021, 1.367)	**0.025**	1.030 (0.844, 1.257)	0.722	1.341 (1.070, 1.681)	**0.011**
Model 2	1.006 (0.842, 1.201)	0.947	0.890 (0.760, 1.122)	0.324	1.284 (1.010, 1.632)	**0.042**
Obesity^†^						
Model 1	1.783 (1.239, 2.566)	**0.002**	1.171 (0.764, 1.793)	0.469	3.006 (1.488, 6.071)	**0.002**
Model 2	1.647 (1.133, 2.395)	**0.009**	1.333 (0.865, 2.054)	0.192	3.333 (1.641, 6.772)	**0.001**
TG, per increase of 1 mmol/L						
Model 1	1.349 (1.126, 1.617)	**0.001**	1.188 (0.918, 1.537)	0.190	1.671 (1.300,2.147)	**<0.001**
Model 2	1.312 (1.087, 1.583)	**0.005**	1.092 (0.839, 1.422)	0.513	1.618 (1.254,2.087)	**<0.001**
TC, per increase of 1 mmol/L						
Model 1	1.025 (0.879, 1.195)	0.754	0.950 (0.790, 1.143)	0.588	1.430 (1.094, 1.869)	**0.009**
Model 2	1.149 (0.986, 1.340)	0.076	1.039 (0.862, 1.253)	0.685	1.356 (1.022, 1.799)	**0.035**
LDL-C, per increase of 1 mmol/L						
Model 1	0.892 (0.714, 1.074)	0.227	0.810 (0.645, 1.016)	0.068	1.200 (0.878, 1.640)	0.253
Model 2	0.979 (0.813, 1.180)	0.825	0.866 (0.689, 1.089)	0.218	1.268 (0.924, 1.739)	0.142
HDL-C, per increase of 0.5 mmol/L						
Model 1	0.900 (0.749, 1.082)	0.274	0.935 (0.753, 1.162)	0.547	0.904 (0.644, 1.268)	0.557
Model 2	1.032 (0.857, 1.244)	0.737	1.081 (0.863, 1.353)	0.301	0.928 (0.661, 1.304)	0.668

*Note*: IFG, impaired fasting glucose; TC, total cholesterol; TG, triglyceride; HDL-C, high-density cholesterol; LDL-C, low-density cholesterol; OR, odds ratios; ^†^Wasting patients were not included for analysis.  ^*∗*^Assessed by logistic regression. Model 1: crude OR; Model 2: age and sex were adjusted for in Model 2 when analyzing the associations of potential influencing factors with IFG in total participants. However, in subgroup analyses on the females and males, only age was adjusted for in Model 2. *P* values <0.05 are presented in bold.

## Data Availability

Due to the sensitive nature of the questions asked in this study, participants were assured raw data would remain confidential and would not be shared.

## Supplementary Materials

Supplementary Materials Table S1: dietary factors and physical activities stratified by different statuses of FPG.
