# Supplementary material for: The Prevalence and Associated Factors of Impaired Fasting Glucose among Children and Adolescents in Urban China: A Large-Scale Cross-Sectional Study
Source: Pediatr Diabetes. 2024 Mar 15;2024:6701192. doi: 10.1155/2024/6701192 (PMC12017157; doi:10.1155/2024/6701192)
Supplement: Supplementary Materials — Table S1: dietary factors and physical activities stratified by different statuses of FPG. [file 6701192.f1.docx]

**Supplemental materials**

**Expanded result:**

**Association of fasting glucose with physical activities and dietary intakes**

Sugar-sweetened beverages intake was observed significantly higher in those with IFG compared to normal participants (*p* < 0.05), as displayed in **Table S1**. No significant difference of physical activity was found between normal participants and those with IFG.

| **Table S1.** Dietary factors and physical activities stratified by different status of FPG^†^ | | | |
| --- | --- | --- | --- |
| **Variables** | **Participants with normal FPG** | **Participants with IFG** | ***p* values*^*^*** |
|  |  |  |  |
| **Physical activity (minutes/day, M±SD)** | | | |
| Vigorous intensity activities | 30.27 ± 49.22 | 30.27 ± 38.05 | 0.998 |
| Moderate intensity activities | 29.73 ± 43.88 | 27.41 ± 28.74 | 0.431 |
| Walking | 46.95 ± 63.51 | 39.20 ± 53.10 | 0.071 |
| Sedentary behavior | 349.92 ± 228.18 | 365.45 ± 222.69 | 0.334 |
| **Dietary factors (servings/day, M±SD)** | | | |
| Fruit intake | 1.32 ± 1.03 | 1.24 ± 1.10 | 0.250 |
| Vegetables intake | 1.86 ± 1.41 | 1.81 ± 1.35 | 0.535 |
| Meat intake | 1.28 ± 1.25 | 1.18 ± 1.08 | 0.200 |
| Sugar-sweetened beverages intake | 0.39 ± 0.69 | 0.50 ± 0.74 | **0.024** |

Note: FPG, fasting plasma glucose; IFG, impaired fasting glucose.

**^†^** Status of FPG including normal FPG and IFG.

**^*^** Participants with IFG vs. participants with normal FPG, *t*-test for continuous variables and *Chi-square* test for categorical variables.
